# Supplementary material for: Pyruvate Dehydrogenase Kinase 1 inhibition mediated oxidative phosphorylation enhancement in cartilage promotes osteoarthritis progression
Source: BMC Musculoskelet Disord. 2023 Jul 20;24:597. doi: 10.1186/s12891-023-06585-6 (PMC10357736; doi:10.1186/s12891-023-06585-6)
Supplement: Supplementary file 1 — Additional file 1. [file 12891_2023_6585_MOESM1_ESM.zip › BP.Gsea.1653623667859/GOBP_PROTEIN_INSERTION_INTO_MEMBRANE.html]

Details for gene set GOBP\_PROTEIN\_INSERTION\_INTO\_MEMBRANE[GSEA]

|  || Dataset | 55457\_collapsed\_to\_symbols.cls.cls #OA\_versus\_normal.cls.cls #OA\_versus\_normal\_repos |
| Phenotype | cls.cls#OA\_versus\_normal\_repos |
| Upregulated in class | OA |
| GeneSet | GOBP\_PROTEIN\_INSERTION\_INTO\_MEMBRANE |
| Enrichment Score (ES) | 0.40704617 |
| Normalized Enrichment Score (NES) | 1.6523216 |
| Nominal p-value | 0.0076530613 |
| FDR q-value | 0.21354926 |
| FWER p-Value | 1.0 |
Table: GSEA Results Summary

  

Fig 1: Enrichment plot: GOBP\_PROTEIN\_INSERTION\_INTO\_MEMBRANE      
 Profile of the Running ES Score & Positions of GeneSet Members on the Rank Ordered List

  

| SYMBOL | TITLE | RANK IN GENE LIST | RANK METRIC SCORE | RUNNING ES | CORE ENRICHMENT || 1 | SAMM50 | SAMM50 sorting and assembly machinery component [Source:HGNC Symbol;Acc:HGNC:24276] | 99 | 0.915 | 0.0465 | Yes |
| 2 | GET1 | guided entry of tail-anchored proteins factor 1 [Source:HGNC Symbol;Acc:HGNC:12790] | 159 | 0.852 | 0.0924 | Yes |
| 3 | NDUFA13 | NADH:ubiquinone oxidoreductase subunit A13 [Source:HGNC Symbol;Acc:HGNC:17194] | 236 | 0.795 | 0.1336 | Yes |
| 4 | WDR83OS | WD repeat domain 83 opposite strand [Source:HGNC Symbol;Acc:HGNC:30203] | 239 | 0.791 | 0.1804 | Yes |
| 5 | EMC9 | ER membrane protein complex subunit 9 [Source:HGNC Symbol;Acc:HGNC:20273] | 276 | 0.768 | 0.2231 | Yes |
| 6 | UBL4A | ubiquitin like 4A [Source:HGNC Symbol;Acc:HGNC:12505] | 464 | 0.675 | 0.2486 | Yes |
| 7 | EMC7 | ER membrane protein complex subunit 7 [Source:HGNC Symbol;Acc:HGNC:24301] | 468 | 0.673 | 0.2882 | Yes |
| 8 | TRAM1 | translocation associated membrane protein 1 [Source:HGNC Symbol;Acc:HGNC:20568] | 569 | 0.633 | 0.3180 | Yes |
| 9 | EMC1 | ER membrane protein complex subunit 1 [Source:HGNC Symbol;Acc:HGNC:28957] | 1067 | 0.502 | 0.3090 | Yes |
| 10 | EMC2 | ER membrane protein complex subunit 2 [Source:HGNC Symbol;Acc:HGNC:28963] | 1096 | 0.497 | 0.3363 | Yes |
| 11 | GET3 | "guided entry of tail-anchored proteins factor 3, ATPase [Source:HGNC Symbol;Acc:HGNC:752]" | 1161 | 0.485 | 0.3601 | Yes |
| 12 | MOAP1 | modulator of apoptosis 1 [Source:HGNC Symbol;Acc:HGNC:16658] | 1215 | 0.473 | 0.3840 | Yes |
| 13 | RTP4 | receptor transporter protein 4 [Source:HGNC Symbol;Acc:HGNC:23992] | 1273 | 0.463 | 0.4070 | Yes |
| 14 | EMC3 | ER membrane protein complex subunit 3 [Source:HGNC Symbol;Acc:HGNC:23999] | 1624 | 0.410 | 0.4041 | No |
| 15 | TIMM8B | translocase of inner mitochondrial membrane 8 homolog B [Source:HGNC Symbol;Acc:HGNC:11818] | 1877 | 0.376 | 0.4068 | No |
| 16 | TIMM10 | translocase of inner mitochondrial membrane 10 [Source:HGNC Symbol;Acc:HGNC:11814] | 2244 | 0.333 | 0.3981 | No |
| 17 | EMC6 | ER membrane protein complex subunit 6 [Source:HGNC Symbol;Acc:HGNC:28430] | 2839 | 0.266 | 0.3677 | No |
| 18 | MAL | "mal, T cell differentiation protein [Source:HGNC Symbol;Acc:HGNC:6817]" | 3265 | 0.224 | 0.3479 | No |
| 19 | TIMM22 | translocase of inner mitochondrial membrane 22 [Source:HGNC Symbol;Acc:HGNC:17317] | 3355 | 0.216 | 0.3538 | No |
| 20 | AGK | acylglycerol kinase [Source:HGNC Symbol;Acc:HGNC:21869] | 3373 | 0.214 | 0.3652 | No |
| 21 | REEP1 | receptor accessory protein 1 [Source:HGNC Symbol;Acc:HGNC:25786] | 3589 | 0.195 | 0.3600 | No |
| 22 | CAMLG | calcium modulating ligand [Source:HGNC Symbol;Acc:HGNC:1471] | 3668 | 0.188 | 0.3651 | No |
| 23 | MAIP1 | matrix AAA peptidase interacting protein 1 [Source:HGNC Symbol;Acc:HGNC:26198] | 4217 | 0.136 | 0.3305 | No |
| 24 | TOMM70 | translocase of outer mitochondrial membrane 70 [Source:HGNC Symbol;Acc:HGNC:11985] | 4553 | 0.109 | 0.3109 | No |
| 25 | TIMM9 | translocase of inner mitochondrial membrane 9 [Source:HGNC Symbol;Acc:HGNC:11819] | 4603 | 0.104 | 0.3133 | No |
| 26 | TRAM2 | translocation associated membrane protein 2 [Source:HGNC Symbol;Acc:HGNC:16855] | 4913 | 0.079 | 0.2939 | No |
| 27 | BAG6 | BAG cochaperone 6 [Source:HGNC Symbol;Acc:HGNC:13919] | 5023 | 0.071 | 0.2897 | No |
| 28 | SEC61A1 | SEC61 translocon subunit alpha 1 [Source:HGNC Symbol;Acc:HGNC:18276] | 5378 | 0.041 | 0.2646 | No |
| 29 | TOMM22 | translocase of outer mitochondrial membrane 22 [Source:HGNC Symbol;Acc:HGNC:18002] | 5391 | 0.041 | 0.2661 | No |
| 30 | TIMM10B | translocase of inner mitochondrial membrane 10B [Source:HGNC Symbol;Acc:HGNC:4022] | 5543 | 0.029 | 0.2560 | No |
| 31 | OXA1L | OXA1L mitochondrial inner membrane protein [Source:HGNC Symbol;Acc:HGNC:8526] | 5974 | -0.003 | 0.2228 | No |
| 32 | TIMM13 | translocase of inner mitochondrial membrane 13 [Source:HGNC Symbol;Acc:HGNC:11816] | 6090 | -0.012 | 0.2145 | No |
| 33 | EMC8 | ER membrane protein complex subunit 8 [Source:HGNC Symbol;Acc:HGNC:7864] | 6104 | -0.013 | 0.2143 | No |
| 34 | GZMB | granzyme B [Source:HGNC Symbol;Acc:HGNC:4709] | 6132 | -0.015 | 0.2131 | No |
| 35 | TIMM8A | translocase of inner mitochondrial membrane 8A [Source:HGNC Symbol;Acc:HGNC:11817] | 7138 | -0.092 | 0.1403 | No |
| 36 | BCS1L | "BCS1 homolog, ubiquinol-cytochrome c reductase complex chaperone [Source:HGNC Symbol;Acc:HGNC:1020]" | 7578 | -0.125 | 0.1136 | No |
| 37 | NMT1 | N-myristoyltransferase 1 [Source:HGNC Symbol;Acc:HGNC:7857] | 7758 | -0.138 | 0.1078 | No |
| 38 | MAPK8 | mitogen-activated protein kinase 8 [Source:HGNC Symbol;Acc:HGNC:6881] | 8956 | -0.228 | 0.0282 | No |
| 39 | PDCD5 | programmed cell death 5 [Source:HGNC Symbol;Acc:HGNC:8764] | 9039 | -0.235 | 0.0358 | No |
| 40 | BAX | "BCL2 associated X, apoptosis regulator [Source:HGNC Symbol;Acc:HGNC:959]" | 9323 | -0.257 | 0.0290 | No |
| 41 | HSPA4 | heat shock protein family A (Hsp70) member 4 [Source:HGNC Symbol;Acc:HGNC:5237] | 9797 | -0.301 | 0.0101 | No |
| 42 | SGTA | small glutamine rich tetratricopeptide repeat co-chaperone alpha [Source:HGNC Symbol;Acc:HGNC:10819] | 11185 | -0.455 | -0.0708 | No |
| 43 | EMC10 | ER membrane protein complex subunit 10 [Source:HGNC Symbol;Acc:HGNC:27609] | 11497 | -0.501 | -0.0653 | No |
| 44 | EGFR | epidermal growth factor receptor [Source:HGNC Symbol;Acc:HGNC:3236] | 12109 | -0.622 | -0.0760 | No |
| 45 | BBC3 | BCL2 binding component 3 [Source:HGNC Symbol;Acc:HGNC:17868] | 12298 | -0.676 | -0.0505 | No |
| 46 | CCDC47 | coiled-coil domain containing 47 [Source:HGNC Symbol;Acc:HGNC:24856] | 12495 | -0.746 | -0.0215 | No |
| 47 | HSP90AA1 | heat shock protein 90 alpha family class A member 1 [Source:HGNC Symbol;Acc:HGNC:5253] | 12730 | -0.893 | 0.0132 | No |
Table: GSEA details [plain text format]

  

Fig 2: GOBP\_PROTEIN\_INSERTION\_INTO\_MEMBRANE      
 Blue-Pink O' Gram in the Space of the Analyzed GeneSet

  

Fig 3: GOBP\_PROTEIN\_INSERTION\_INTO\_MEMBRANE: Random ES distribution      
 Gene set null distribution of ES for **GOBP\_PROTEIN\_INSERTION\_INTO\_MEMBRANE**
